# Inductive Thermal Effect of Ferrite Magnetic Nanoparticles

**DOI:** 10.3390/ma12193208

**Published:** 2019-09-30

**Authors:** Jeotikanta Mohapatra, Meiying Xing, J. Ping Liu

**Affiliations:** Department of Physics, University of Texas at Arlington, Arlington, TX 76019, USA; jeotikanta.mohapatra@uta.edu (J.M.); meiying.xing@mavs.uta.edu (M.X.)

**Keywords:** iron oxide, magnetic nanoparticles, magnetic nanorods, nanocrystals nanoassemblies, superparamagnetism, colloidal stability, hyperthermia

## Abstract

Localized heat induction using magnetic nanoparticles under an alternating magnetic field is an emerging technology applied in areas including, cancer treatment, thermally activated drug release and remote activation of cell functions. To enhance the induction heating efficiency of magnetic nanoparticles, the intrinsic and extrinsic magnetic parameters influencing the heating efficiency of magnetic nanoparticles should be effectively engineered. This review covers the recent progress in the optimization of magnetic properties of spinel ferrite nanoparticles for efficient heat induction. The key materials factors for efficient magnetic heating including size, shape, composition, inter/intra particle interactions are systematically discussed, from the growth mechanism, process control to chemical and magnetic properties manipulation.

## 1. Introduction

**Figure 1:**
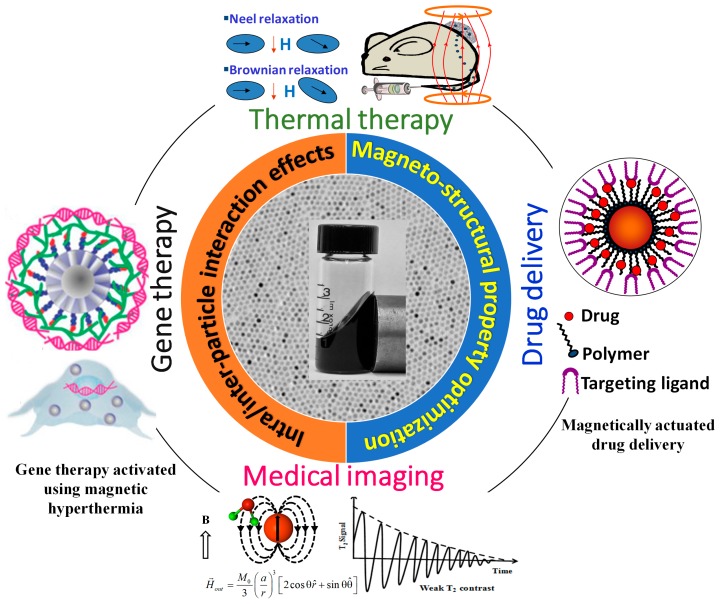
Based on the heat activation ability, these nanostructures have been utilized in cancer therapy, on-demand drug carrier and gene therapy applications [32,33]. The magnetic nanoparticles have also been as contrast agent in magnetic particle imaging (MPI) and magnetic resonance imaging (MRI).

Ferrite (MFe_2_O_4_, M = Mn, Fe, Co, Ni and Zn) magnetic nanoparticles (typically, of size 5–100 nm) have attracted profuse attention because they are at the interfaces of chemistry, physics and biology, due to their remarkable size and shape-dependent magnetic properties [1,2,3]. Scientists have developed methods to produce magnetic nanoparticles with fine control of the morphology [4,5,6]. Many new phenomena such as superparamagnetism, superferromagnetism, and superspin glass have been observed in these magnetic nanoparticles (MNPs) [7,8,9]. Moreover, in the nano-regime the magnetic properties such as coercivity (*H_C_*), saturation magnetization (*M_S_*) and susceptibility (*χ*) strongly vary with the size, shape, and composition of the magnetic nanoparticles [2,6,10]. These unique magnetic properties, small size and biocompatibility make them particularly promising in various biomedical applications, for instance contrast enhancement in magnetic resonance imaging (MRI), nano-sized carrier in drug delivery, mediators in converting electromagnetic energy to heat, and as magnetic-targeting and bio-sensing agents [11,12,13,14,15]. All these biomedical applications require the MNPs to have high magnetic moments with small sizes and a narrow particle-size distribution, so that the nanoparticles could have well-defined physical and chemical properties [11,16,17,18]. A general picture in Figure 1 schematically illustrates the uses of MNPs in four important areas of cancer theranostics.

In general, when the magnetic nanoparticle suspension is subjected to an alternating current magnetic field (ACMF), it can demonstrate prominent heating effects related to energy losses during the magnetization reversal of the nanostructures. This heating ability depends on the properties of the nanostructures, such as mean size, magnetization and magnetic anisotropy, and the ACMF amplitude (*H_ac_*) and frequency (*f*) [19,20]. Therefore, to improve the inductive heating characteristics of magnetic nanostructures, many approaches have been taken to improve the magnetic properties of the nanostructures [21,22,23,24,25]. Over the past few years, substantial progress has been made to precisely control the size, composition, morphology and multifunctionalities of magnetic nanostructures [11,26,27,28,29]. Such studies have improved our understanding of their unique nanoscale magnetic properties and demonstrated the great potential of these MNPs in hyperthermia applications. For example, MNPs with hard and soft ferrite core-shell architecture exhibit strong exchange-coupling, which enhances the magnetic heating power [30,31]. The magnetic nanostructure comprises of several domains such as nanoflowers, nanoclusters and nanoassemblies have also been shown to enhance the heating power [24,25]. In this review, we begin by briefly introducing the fundamental principles of MNPs. We then focus on the chemical synthesis, in particular the organic phase synthesis, of a series of MNPs with controlled size, composition, and morphology. Finally, we establish a relationship across the magnetic and inductive heating properties of MNPs.

## 2. Characteristics of Magnetic Nanoparticles and Inductive Heating Principles

### 2.1. Magnetism at Nanoscale

**Figure 2:**
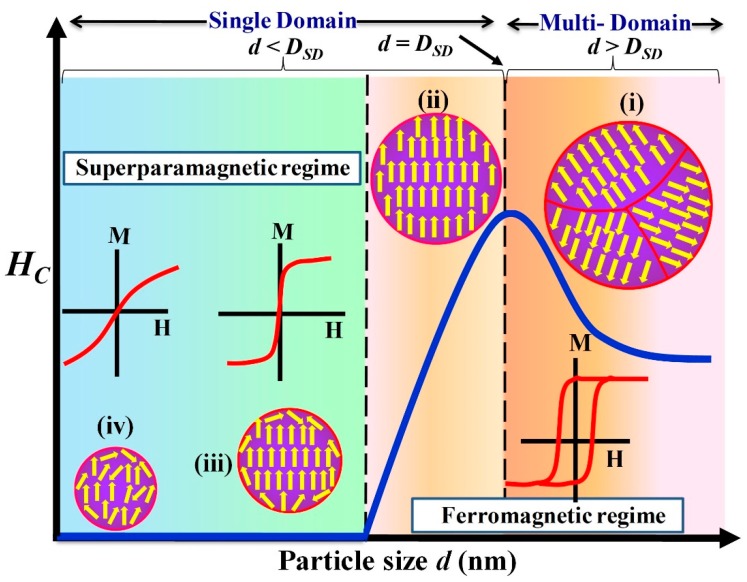
Variation of coercivity (*H_C_*), spin ordering and the nature of the magnetization curve with MNPs size ‘*d*’. (i) With a decrease of the size of ferromagnetic particles, the *H_C_* initially increases and then attains a maximum value at the critical single domain size (*D_SD_*) due to coherent rotation. (ii) Further, in the single domain regime (*d* < *D_SD_*), the *H_C_* decreases as the particle size decreases and (iii) becomes zero at the superparamagnetic regime due to the thermal agitation. (iv) Finally, in ultra-small regimes (*d* ≤ 3 nm), due to high-spin disorder of elemental spins, the MNPs exhibit a linear relationship between the magnetization and the applied magnetic field [35].

**Figure 3:**
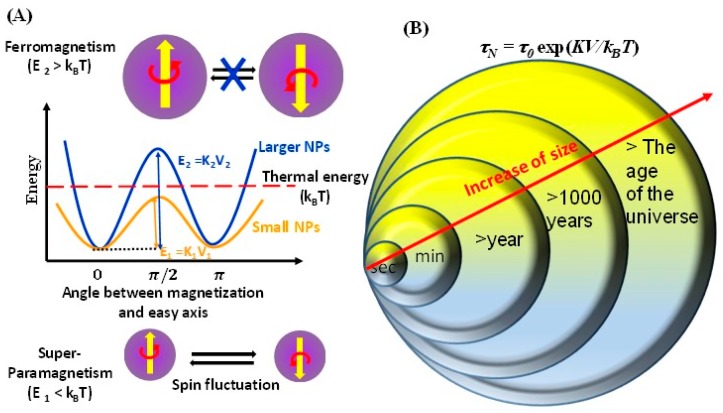
(**A**) The top of (A) depicts the reversal of magnetization in ferromagnetic particles. The energy diagram below illustrates the difference in energy barrier for a large particle behaving as ferromagnet and a small particle behaving as superparamagnet. The bottom of (A) depicts the relaxation process in superparamagnetic particles. (**B**) A typical correlation between the Néel relaxation time and the MNPs diameters. MNPs with extremely high *τ_N_* are promising for recording media applications.

As first predicted by Frenkel and Dorfman, when the size of the ferromagnet or ferrimagnet decreases below a critical size, the amount of energy required to produce domain walls becomes greater than the reduction in the magnetostatic energy [34]. As a consequence, only single domain formation is favored. A single domain particle consists of large numbers of atomic spins and, thus, can be viewed as ‘supermoment’ that has a magnetic moment ~10^3^ to 10^5^ *µ_B_* [35]. The single domain size can be estimated from the equation [34,35,36]:

(1) $$D_{SD}=\frac{18{\left(AK\right)}^{\frac{1}{2}}}{\mu_{0}M_{S}{}^{2}}$$

where μ_0_ is the permeability of free space (4π × 10^−7^ H/m), *A* is the exchange stiffness in J/m and *K* is the magnetocrystalline anisotropy in J/m^3^. For example, the typical value of single domain size is around 128 nm for Fe_3_O_4_, while for MnFe_2_O_4_ it is 50 nm (due to a smaller *K* and a higher *M_S_*) [37,38,39]. Further, at the particle size *d* = *D_SD_*, the single domain prefers to be uniformly magnetized along one of its anisotropic easy axes, which leads to a strong enhancement in coercivity. Below *D_SD_*, due to the decrease of the magnetic anisotropy energy (*E_a_* = *KV*, *V* is volume of the particle) the coercivity value decreases with decrease in the size (see Figure 2) [40]. On further reduction in size, the anisotropy energy value decreases further and becomes comparable to or even lower than the thermal energy (*k_B_T*, *k_B_* is Boltzmann constant). As a consequence, the energy barrier for magnetization reversal is dominated by thermal energy (Figure 3A, orange line). Thus, the supermoment thermally fluctuate like spins in a paramagnetic material, which leads to a net magnetization of zero; this phenomenon is called superparamagnetism. Superparamagnetic materials have no coercivity at room temperature, whereas ferromagnetic materials have a high coercivity. On application of an external magnetic field, the superparamagnetic nanoparticles react like the paramagnets (i.e., supermoment rotation) with the one exception that their magnetic susceptibility ‘*χ*’ is much larger or even comparable to bulk value. However, in ultra-small sized MNPs, the *χ* value is always lower than the bulk value. This lower value is explained by the magnetically inactive surface atomic layer of the nanoparticles. Since a large fraction of atoms reside at the surface of MNPs, the surface spin disorder effect is dramatically pronounced with decrease of MNPs size. Thus, with a decrease of the size of MNPs, *χ* value decreases. The alternative approach to modulate the magnetic properties (*χ* and *H_C_*) at the nanoscale is to develop the anisotropy through other mechanisms such as the shape anisotropy and the exchange coupling. The effects of shape anisotropy and the exchange coupling on the magnetic properties of MNPs will be disscused in the Section 3.3 and Section 3.4, respectively. 

The superparamagnetic relaxation time can be modeled by the Néel-Brown theory as shown below [41,42]:

(2) $$\tau_{N}=\tau_{0}exp\left[\frac{E_{a}}{k_{B}T}\right]$$

where *E_a_ = KV* is the anisotropy energy which determines the flipping angle of the nanoparticle, and *τ*_0_ is an attempt relaxation time factor that lies in the range of 10^−9^ to 10^−13^ s [43]. The relaxation time exponentially increases with the increase of MNPs size (Figure 3B). When the relaxation time ‘*τ_N_*’ is small or comparable to the time scale of the experimental technique (*τ_N_ ≤ t_m_*, *t_m_* is the measuring time), we measure an average value of the magnetization; however, if the *τ_N_ ≥ t_m_*, we measure the instantaneous value of the magnetization. The transition temperature at which *τ_N_ = t_m_* is known as superparamagnetic blocking temperature (*T_B_*) and can be expressed as

(3) $$T_{B}=\frac{KV}{k_{B}ln\left(t_{m}/\tau_{0}\right)}$$

From the above equation it is clear that the magnetocrystalline anisotropy and the volume of magnetic nanoparticles are the two key parameters on which the *T_B_* depends. The larger the size of the nanoparticles, the larger ‘*k_B_T’* is required for superparamagnetic transition. Thus, *T_B_* increases with the increase in the size of the nanoparticles. However, in the case of nanoparticle assemblies and multicore nanoparticles, the interparticle interaction modifies the energy barrier and produces collective properties. The magnetic properties of interacting MNPs are well described by the Vogel-Fulcher model [44,45]

(4) $$\tau =\tau_{0}exp\left[\frac{E_{a}}{k_{B}\left(T-T_{0}\right)}\right]$$

where *T*_0_ is known as Vogel-Fulcher temperature, a measure of the interaction strength and *E*_a_/*k*_B_ is the activation energy required to overcome the barrier of the reversal of the magnetization. With a further increase of nanoparticle interaction, a spin-glass like collective state can be formed (known as superspin glass) [35,46]. When the strength of the interparticle interactions is strong enough, a long-range ferromagnetic ordering can occur, and the state is known as superferromagnet [41,42]. Recently, the superspin glass and superferromagnetic ordering have been observed in Fe_3_O_4_ and Co nanoparticle systems [47,48]. As discussed above the magnetic properties of MNPs can be controlled with exploitation of several parameters such as size, composition and morphology. In addition, the inter/intra particles interactions can be controlled to further optimize the magnetic properties, which will be discussed in the Section 3.4. 

### 2.2. Basic Principles of Inductive Heating 

#### 2.2.1. Effects of Magnetic Anisotropy and Magnetic Susceptibility 

**Figure 4:**
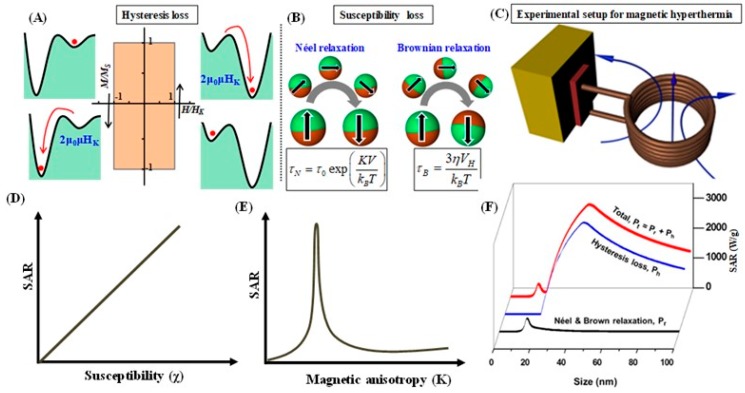
(**A**) Schematic view of the hysteresis loss in a ferromagnetic nanoparticle. Hysteresis loss in one cycle of ACMF is related to the area of the hysteresis loop and the Zeeman energy in magnetic field [56,57]. (**B**) Susceptibility loss given by the out-of-phase component of AC susceptibility is associated to the nanoparticle’s relaxation. As demonstrated in the schematic, in Néel relaxation, the magnetic moment shown by the black arrow reverses (the particle does not rotate), while in Brownian relaxation, the particle (the sphere) rotates as a whole. (**C**) Schematic view of a typical hyperthermia setup. The variation of SAR based on (**D**) susceptibility (*χ*) and (**E**) magnetic anisotropy (*K*). (**F**) Size dependent SAR resulting from the susceptibility loss and the hysteresis loss [55].

When colloids of magnetic nanoparticles are subjected to an ac magnetic field, they convert electromagnetic energy into heat due to losses from the reversal of magnetization [49,50,51]. The magnetization reversal mainly occurs through two different processes: Hysteresis loss (seen in ferromagnetic NPs) and susceptibility loss (seen in superparamagnetic NPs). Hysteresis loss strongly depends on the field amplitude (*H_ac_*) as well as the magnetic coercive field (*H_K_ =* 2*K/μ_0_M_S_*, however in experiment the coercivity will be much lower than the *H_K_*) [52]. When the ACMF is applied to a ferromagnetic particle with moment (*μ*), the magnetization state becomes metastable, as shown in Figure 4A. Subsequently, *μ* reverses and, therefore, the Zeeman energy falls from *μ_0_μH_K_* to –*μ_0_μH_K_* and the corresponding energy difference dissipates as heat. In the magnetization reversal event, the work done in one cycle of the ACMF ‘*H_ac_sin* (*2πft*)’ is 0 for *H_ac_* << *H_K_* and *4μ_0_μH_K_* for *H_ac_* ≥ *H_K_*. In the latter case, the heat dissipation from MNPs per unit weight during unit time, also known as specific loss power *P_H_* can be expressed as 4*μ_0_μH_K_fφ^−1^* (4*μ_0_M_S_H_K_fρ^−1^*), where *φ* and *ρ* are the weight and density of the MNPs, respectively. In short, the *P_H_* value increases from 0 to 4*μ_0_μH_K_fφ^−1^* with increase of *H_ac_* and flattens out if *H_ac_* is strengthened above *H_K_*. Thus, the guiding principle for maximizing *P_H_* is that *H_ac_* is adjusted to *H_K_* and the number of ACMF cycles is maximized. Unfortunately, because of the technical restrictions on the ACMF amplitude and physiological limitations, the hysteresis loss cannot be completely used. Figure 4C shows the schematic representation of a typical hyperthermia setup. 

In smaller-sized magnetic nanoparticles (*KV* ≤ *k_B_T*), the magnetization reversal of ‘*μ*’ is caused by thermal fluctuation. The magnetization reversal in a zero-magnetic field is associated with Néel relaxation and Brownian rotation of the nanoparticles (Figure 4B) [53]. The Néel (*τ_N_*) and Brownian (*τ_B_*) relaxation times are given by [54]

(5) $$\tau_{N}=\tau_{0}exp\left[\frac{KV}{k_{B}T}\right]$$

(6) $$\tau_{B}=\frac{3\eta V_{H}}{k_{B}T}$$

The Néel relaxation is controlled by anisotropy energy (*KV*) of nanoparticles. Equation (6) for the Brownian rotation is directly related to hydrodynamic parameters such as the hydrodynamic volume (*V_H_*) and the viscosity of the medium (*η*). 

If Néel relaxation and Brownian rotation occur in parallel, the effective relaxation time *τ* is given by [54]

(7) $$\frac{1}{\tau }=\frac{1}{\tau_{B}}+\frac{1}{\tau_{N}}$$

(8) $$\mathrm{Hence},\;\tau =\frac{M_{S}}{2\gamma_{0\;}K^{3/2}}{\left(\frac{\pi k_{B}T}{V}\right)}^{1/2}exp\left(\frac{KV}{k_{B}T}\right)$$

For superparamagnetic nanoparticles, *τ* is determined only by $\tau_{N}$ because it decreases exponentially with decreasing the nanoparticles volume, while the decrease of $\tau_{B}$ is directly proportional to *V_H_*. Further, for small *H_ac_*, a linear response of the thermodynamic equilibrium state of the superparamagnetic nanoparticles (i.e., ACMF driven reversals) can be considered. In this case, the average of out-of-phase component of AC susceptibility $\;\chi^{\prime \prime }$ can be expressed as follows,

(9) $$\chi^{\prime \prime }=\frac{\mu_{0}\mu^{2}}{3k_{B}T}\times \frac{\omega \tau }{1+{\left(\omega \tau \right)}^{2}}$$

Consequently, the susceptibility loss occurs, and the corresponding specific loss power *P* is expressed as [54].

(10) $$P=\pi \mu_{0}\chi^{\prime \prime }.H_{ac}^{2}\;.f.\rho^{-1}=\frac{\pi \mu_{0}^{2}\mu^{2}H_{ac}^{2}}{3\rho k_{B}T}\frac{2\pi f^{2}\tau }{\left[1+{\left(2\pi f\tau \right)}^{2}\right]}$$

Equation (10) indicates that *P* increases in proportion to *f*^2^ in the low frequency range  2πf≪τ, whereas, it flattens out at $\pi \mu_{0}^{2}\mu^{2}H_{ac}^{2}/3\rho k_{B}T\;$ even though *f* is increased further in the high frequency range  2πf≫τ. Thus, the guiding principle for maximizing the specific loss power in the superparamagnetic regime is that *f* can be fine-tuned to $\;\tau^{-1}$ and *H_ac_* can be maximized (Figure 4D–F). Further, from the above equation 8 and 10, it is evident that the increased *M_S_* values lead to a higher *P* and hence metallic nanoparticles including Fe, Co and FeCo (*M_S_* in the range of 150–220 emu/g) are suitable heating mediators, but they have not been considered for their cytotoxicity and poor stability in physiological environment. The heating activity of MNPs also need fine-tuning of the magnetic anisotropy constant *K*, it shifts the critical MNPs size that corresponds to the maximum *P* to a lower value. However, smaller-sized MNPs possess the low moment and, hence they can exhibit poor heating activities. In short, an ideal material in the superparamagnetic regime would have a high *M_S_* and intermediate *K* value.

The amount of heat generated by MNPs is usually quantified in terms of the specific absorption rate (SAR), given by [21]

(11) $$SAR=\frac{C}{m}\left(\frac{dT}{dt}\right)$$

where *C* is the specific heat of the colloid (i.e., for water, this value is 4.18 J g^−1^ C^−1^), d*T*/d*t* is the initial slope of the temperature versus time graph, and *m* is mass of magnetic material (mg/ml) in the suspension. As such, both the susceptibility loss and the hysteresis loss are strongly dependent on the MNPs size, and the SAR can show two maxima as shown in Figure 4F [55]. The 1^st^ peak in the superparamagnetic regime is related to the resonance condition ($2\pi f\sim \;\tau^{-1}$) and 2^nd^ peak in the ferromagnetic regime is attributed to the hysteresis loss. In addition, in both regimes a high *M_S_* is required to further optimize the SAR values, which will be discussed later in the Section 3. 

#### 2.2.2. Effect of Curie Temperature (*T_C_*)

One of the most important restrictions of magnetic hyperthermia treatment is the risk of overheating in healthy tissues. A direct approach for overcoming this issue is to make the nanoparticles self-controlled, which can be realized by taking advantage of the MNPs’ Curie temperature (*T_C_*) and adjusting the *T_C_* to the therapeutic temperature (42−45 °C). When the temperature increases above the *T_C_*, the MNPs lose their ferromagnetic properties and instead shows paramagnetic behavior. Thus, the induction heating is immediately suppressed above the *T_C_*. Recently, this phenomenon has been used to formulate ferrite-based self-controlled heating mediators, mainly the mixed ferrites such as Zn_x_Fe_3-x_O_4_, MnZnFe_2_O_4_, MgFe_2_O_4_, etc. [58,59,60,61]. 

The Curie temperature of a magnetic spinel ferrite can be assessed by the strength of exchange interactions between magnetic moments in the octahedral (*O_h_*) and the tetrahedral (*T_d_*) sublattices. The exchange interactions in ferrites are mediated by intermediating oxygen *p*-orbitals, known as super-exchange interactions. Thus, the *T_C_* can be adjusted by controlling compositions (i.e., magnetic moments of cations in two sublattices) and the distance between cations ions. Table 1 lists several spinel ferrite nanoparticles in which *T_C_* is adjusted by alteration in chemical composition via doping suitable amount of magnetic or nonmagnetic elements. Despite having the *T_C_* in the therapeutic temperature range, a high *M_S_* and moderate *K* are required to obtain high SAR values. Thus, only a few of the ferrites (Zn_x_Fe_3-x_O_4_, MnZnFe_2_O_4_, MgFe_2_O_4_) have shown promising heating activities after adjusting their Curie temperature values to the required therapeutic limit. 

## 3. Enhancing Inductive Heating by Engineering the Magnetic Nanostructures

### 3.1. Size Effects

**Figure 5:**
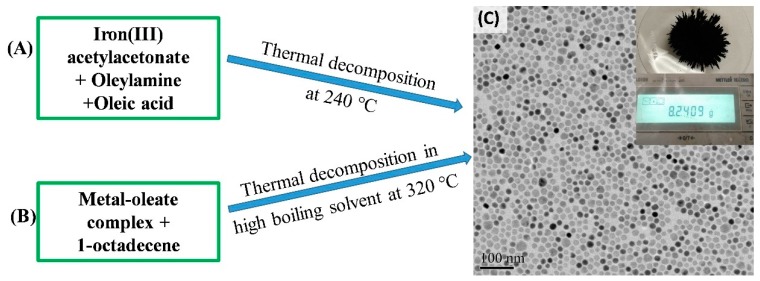
Schematic illustration of two simple approaches have been widely used to synthesize monodispersed iron oxide NPs: (**A**) Thermal decomposition of iron-precursor in oleylamine and oleic acid as surfactant and (**B**) thermal decomposition of metal-organic complex in octadecene. (**C**) Representative TEM image of 12 nm Fe_3_O_4_ NPs prepared in large scale from the decomposition of iron (III) acetylacetonate. The inset is a digital photograph showing 8.2 g of the Fe_3_O_4_ NPs produced in a single batch [71].

**Figure 6:**
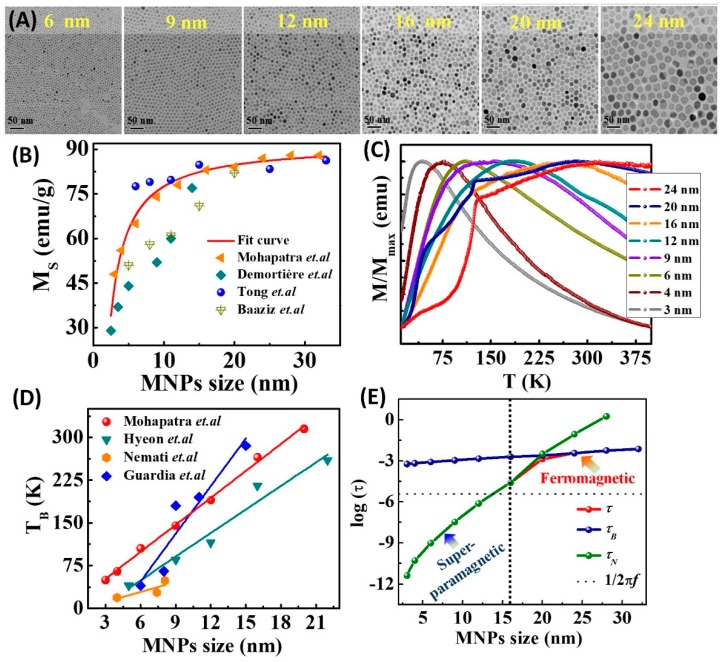
(**A**) The TEM micrographs of differently sized Fe_3_O_4_ nanoparticles. (**B**) A relationship between *M_S_* value and the nanoparticle size [71,76,77,78]. (**C**) The zero-field cooled (ZFC) magnetization curves of different sized Fe_3_O_4_ nanoparticles [71]. (**D**) Blocking temperature (*T_B_*) versus nanoparticle size ‘*d’* [33,71,72]. (**E**) The variation of Neel relaxation time and Brownian relaxation time with Fe_3_O_4_ nanoparticles size [71].

In the past few years, significant attention has been devoted to synthesizing monodispersed magnetic nanoparticles with precise control over their size, composition and shape. While several synthetic strategies have been proposed to synthesize ferrite nanoparticles, the high-temperature solution phase methods appear to be the most interesting because it permits a good control over the size and the morphology of the nanoparticles [33,64]. In this approach, the high-temperature reaction of iron (III) acetylacetonate or iron oleate in the presence of oleic acid, and/or oleylamine leads to the formation of Fe_3_O_4_ nanoparticles [65,66]. A brief pictorial detail of the synthesis procedure is shown in Figure 5. An exciting development in the synthesis of monodispersed iron oxide NPs was demonstrated by Park et al. [33]. In a typical reaction, Fe-oleate complex was prepared by reacting iron chloride with sodium oleate, which was thermally decomposed into monodispersed Fe_3_O_4_ NPs in octadecene at 320 °C. With the help of the seed-mediated growth, Fe_3_O_4_ NPs with 4 to 13 nm diameters were obtained [64]. Later, Xu et al. found that by changing the heating conditions, and ratios of oleylamine and oleic acid, the size of Fe_3_O_4_ NPs can be tuned from 14 to 100 nm [67]. Recently, oleylamine was used as a multitasking agent, acting as a solvent, and reducing and surface functionalizing agents, to prepare monodispersed Fe_3_O_4_ NPs with a reasonably large magnetization value [68,69,70,71]. The experiment confirms that the presence of excess amount of oleylamine offers an adequately strong reductive environment for the Fe-precursor and leads to the formation of Fe_3_O_4_ NPs at a reasonably low temperature of 240 °C. Figure 6A shows the representative TEM micrographs of monodispersed Fe_3_O_4_ nanoparticles of size ranging from 6 to 24 nm, prepared by thermolysis of Iron(III) acetylacetonate in oleic acid and oleylamine [71]. These synthesis methods were also extended to formulate MFe_2_O_4_ (M = Co, Ni, and Mn) NPs. 

The size-dependent characteristics of MNPs are magnetization and blocking temperature, and their values increase with the increase of NPs size (Figure 6). An inherent characteristic of superparamagnetic nanoparticles is that they possess a magnetically disordered spin structure at the surface, because of the reduced exchange coupling between spins at the surface (due to the cations vacancy, ligands and high surface energy). Thus, magnetic nanoparticles are considered to be core-shell structures that are composed of a magnetically ordered core and a disordered shell that is known as spin canting/spin disorder [72,73]. The magnetization as a function of nanoparticles size ‘*d’* and disordered surface layer thickness ‘*t*’ is described as $M_{S}=M_{Bulk}{\left[1-\left(6t/d\right)\right]}^{}$ [74]. The size effect on the *M_S_* value has been observed in both ferrimagnetic and ferromagnetic nanoparticles. Figure 6B shows sharp increases of *M_S_* value with increase in the size of Fe_3_O_4_ nanoparticles prepared via thermal decomposition of Fe-precursor. The calculated spin disorder layer thickness is around 0.2 to 0.5 nm. However, the surface spin disorder effect increases with decreasing the particle size due to the higher surface-to-volume ratio. Therefore, the *M_s_* value approaches to the bulk value (92 emu g^−1^) with the increase of Fe_3_O_4_ NPs size [75].

The blocking behavior in MNPs is observed when the thermal energy (*k_B_T*) exceeds the anisotropy energy [*E_a_ = K_eff_Vsin^2^θ,* where *K_eff_* is the sum of several terms such as magnetocrystalline anisotropy, shape anisotropy, surface anisotropy, and inter-particle coupling between nanoparticles]. Since anisotropy energy is directly proportional to the volume of the nanoparticles, the blocking temperature (*T_B_*) increases with the increase in the size of nanoparticles. The zero-field-cooled magnetization (*M_ZFC_*) curves (at applied field 100 Oe) for the Fe_3_O_4_ nanoparticles are shown in Figure 6C. The obtained *T_B_* values for different sized Fe_3_O_4_ nanoparticles indicates a linear increasing trend of *T_B_* with the size of MNPs as shown in Figure 6D. The effective anisotropy constant can be calculated using the Neél law (*T_B_ = K_eff_V/25k_B_*). The obtained *K_eff_* (1.1 × 10^6^ to 1.5 × 10^4^ J m^−3^) values are much larger than that for bulk magnetite (1.35 × 10^4^ J m^−3^) [40]. The high *K_eff_* value is due to the surface anisotropy contribution from the disordered surface spins layer of the nanoparticles and interparticle interactions between neighbouring nanoparticles. The ZFC magnetization for the 20 and 24 nm sized Fe_3_O_4_ NPs showed a sharp drop at 120 K. This is a characteristic transition of pristine magnetite phase, known as the Verwey transition. Below the Verwey transition temperature, the magnetic easy axis switches from the 〈111〉 to the 〈100〉 direction, which leads to the reduction of *M_ZFC_*. More importantly, the Verwey transition is extremely sensitive to oxidation, in fact it disappears when off-stoichiometry parameter δ, defined as Fe_3(1-δ)_O_4_, is larger than 1% [79].

**Figure 7:**
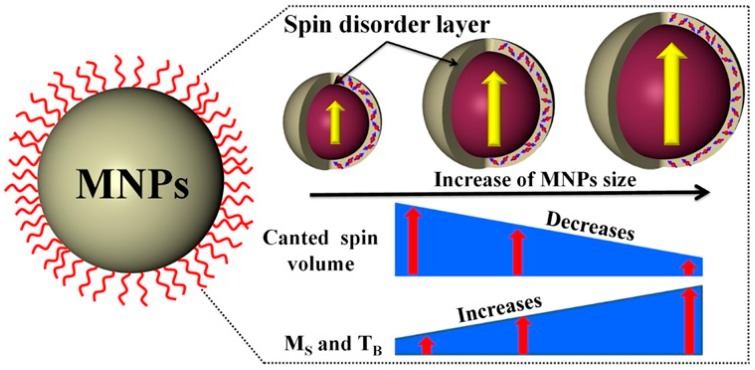
Schematic representation of the variation of spin-canting effect, saturation magnetization, and blocking temperature with the MNPs size.

The Néel relaxation time and the Brownian relaxation time can be calculated using effective magnetic anisotropy constant (*K_eff_*) and the hydrodynamic diameter, respectively. The obtained Brownian, Néel and effective relaxation times for MNPs are plotted as a function of size in Figure 6E. Note that the *τ_N_* value depends more strongly on the MNP size than the *τ_B_* value. Moreover, the *τ_N_* value can be further tuned by controlling the composition of MNPs. Above the superparamagnetic critical size limit, the Brownian relaxation dominates. As the *τ_B_* value is proportional to the *V_H_* of the particles, it can be increased by coating the MNPs with a long-chain surfactant molecule. The scaling relationship between magnetic properties and MNPs size is schematically illustrated in Figure 7. Since the hyperthermia heating efficiency is dependent on the *M_S_* and *K_eff_* of the MNPs, the size control synthesis of MNPs will result in enhanced hyperthermia effects for an optimum size. 

**Figure 8:**
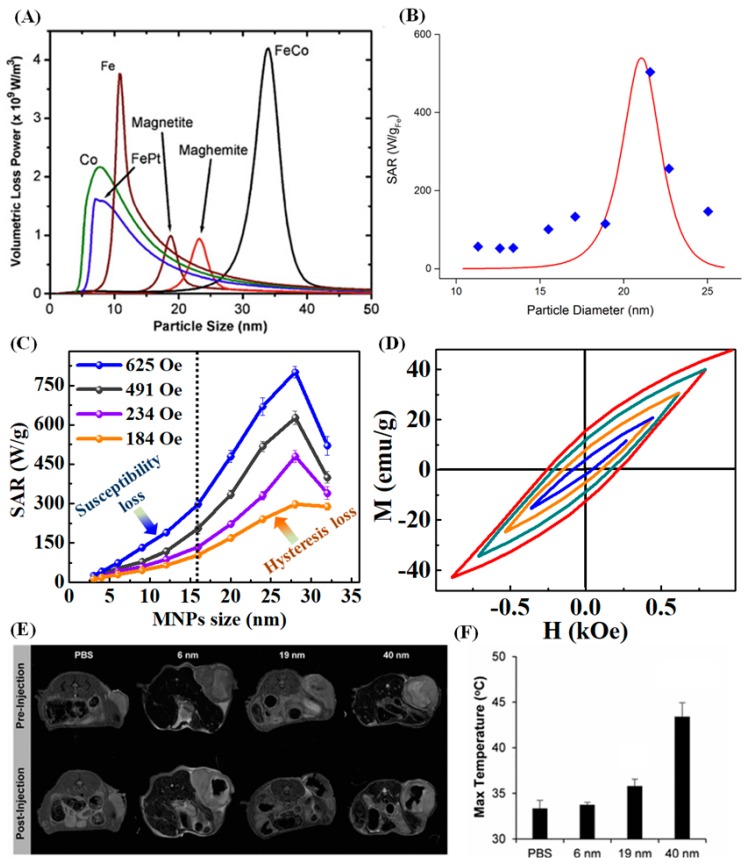
(**A**) Depicts volumetric loss power versus particle size for various MNPs dispersed in water at 10% particle concentration in ACMF (*f* = 300 kHz, *H_ac_* = 50 mT) [80]. (**B**) The theoretical (red) and experimental (blue) SAR values of Fe_3_O_4_ nanoparticles with different diameters [81]. (**C**) Depicts the variation of the SAR values as a function of MNP size at different amplitudes for the ACMF field (184–625 Oe) [71]. (**D**) Shows the room temperature minor M(H) loops of 28 nm MNPs at various magnetic fields: 300 (blue), 500 (orange), 700 (green) and 900 Oe (red) [71]. (**E**) MRI images of the cross section of the mouse bodies before and after infusion of 6, 19 and 40 nm Fe_3_O_4_ nanoparticles dispersion and (F) Maximum temperature reached during 1 h of heating [76].

It is known that according to linear response theory (LRT, Equation (10)), the SAR of superparamagnetic particles is proportional to the applied ACMF amplitude and frequency. Furthermore, the maximum absorption of magnetic energy occurs when the effective relaxation time of MNPs is close to the frequency of the applied ACMF (*ωτ* ≈ 1). This indicates that for a given ACMF frequency (*ω* = 2*πf*) there is an optimal size that resonates well with the applied ACMF. Figure 8A illustrates the size dependency of loss power for different MNPs [80]. As can be seen, the maximum value for heating efficiency occurs at the resonance condition when *ωτ* ≈ 1. Further, the relaxation time *τ* is inversely related to the effective magnetic anisotropy and is determined by the MNPs’ structure. Thus, the critical size corresponding to the maximum loss power is smaller for high magnetic anisotropy materials such as FePt (206 kJ/m^3^), Co (412 kJ/m^3^), and Fe (48 kJ/m^3^) compared to Fe_3_O_4_ (9 kJ/m^3^). In the superparamagnetic regime, Vreeland et al. observed that for an ACMF of *H_ac_* = 36.5 kA/m and *f* = 341 kHz, the optimum size corresponding to maximum SAR is around 22 nm, which matches the theoretical prediction of the LRT (Figure 8B) [81]. Moreover, the heating activity in superparamagnetic regime is mainly ascribed to the Néel relaxation and thus the optimum SAR is achieved at the resonance condition (*ωτ_N_* ≈ 1). Most experimental studies on the size dependency of heating efficiency in the superparamagnetic regime reported that the maximum SAR can be achieved with iron oxide NPs of size ∼15 to 22 nm, depending on the ACMF amplitude and frequency [82,83,84,85,86].

Nevertheless, the recent results are contradictory with the LRT model showing that some of the highest SAR values were observed in MNPs with particle size > 20 nm. Tong et al. have shown that both in the superparamagnetic and ferromagnetic regime the SAR values increased monotonically with nanocrystal size and the most dramatic increases by 50-fold occurred between 11 and 33 nm sized Fe_3_O_4_ nanoparticles [76]. In fact, the maximum SAR values attended are 1026 and 2560 W/g of Fe at 9.35 and 20.7 kA/m field strength, respectively for 40 nm Fe_3_O_4_ nanoparticles. A similar size dependence trend in the SAR values also has been noticed recently by Mohapatra et al. [71]. Figure 8C shows the relative heating performance of Fe_3_O_4_ nanoparticles in the 3–32 nm size range measured under applied ACMF amplitudes of 184, 324, 491 and 625 Oe at a fixed frequency of 265 kHz. It can be seen from the figure that the SAR increases with the increase of nanoparticle size and attains a maximum value at a particle size of 28 nm, then the value decreases with further increase of the particles size. Remarkably, the increasing trend of the SAR has two different scenarios with respect to the MNPs size. The SAR value increases from 27–298 W/g of Fe_3_O_4_ (with *H_ac_* = 625 Oe) with the increases of MNPs size from 3–16 nm and with further increase of the MNPs size it increases rapidly. The comparison between the LRT model prediction and the experimental SAR values (Figure 8B) indicates that the LRT model is applicable only for MNPs size ≤ 16 nm (i.e., the superparamagnetic regime). In theory, increasing the MNPs size above 16 nm can suppress the susceptibility loss due to the particle moment blocking, and consequently the SAR should reduce. Instead, the SAR value increases sharply with the increase of MNPs size from 16–28 nm. The fast increase of the SAR (above 16 nm) values is due to the combined effect of susceptibility loss and hysteresis loss. In the ferromagnetic regime, the amount of dissipated heat as a result of hysteresis loss is proportional to the area under the hysteresis loop, which increases with the increase of MNPs size [87,88,89]. According to the dynamic hysteresis model, the quasi-static hysteresis loops change with the field strength and the frequency of the ACMF. In particular, the hysteresis loop enlarges with the frequency, the magnetic field (Figure 8D) and MNPs size. The improvement of the hysteresis loop area results in the enhanced hysteresis loss in the larger sized MNPs. 

To illustrate the potential applications of ferromagnetic MNPs in cancer treatment, Tong et al., tested 6, 19 and 40 nm MNPs on the mouse xenograft tumor model for glioblastoma multiforme (GBM) [76]. GBM is an aggressive and invasive brain cancer, thus surgical resection will be complicated. An alternative treatment strategy is local hyperthermia treatment. *T*_2_-weighted magnetic resonance (MR) images acquired before and after injection of MNPs (50 μg of Fe) display the sharp contrast of the tumor region (see Figure 8E). A more accurate dosimetry of the spatial temperature profiles during heating treatment are obtained by in vivo quantitative MR imaging of MNPs. Following imaging, the mice are exposed to an ACMF (9.35 kA/m, 325 kHz) for 1 h. The tumors injected with the 6 nm MNPs did not show a temperature increase compared to the control, whereas the 19 and 40 nm MNPs showed a temperature increase of 2.5 and 10.1 °C, respectively (Figure 8F). The 40 nm MNPs are able to reach 43.4 ± 1.5 °C during the course of the treatment, which could be sufficient for many cancer thermal therapies. 

### 3.2. Composition Effects

**Figure 9:**
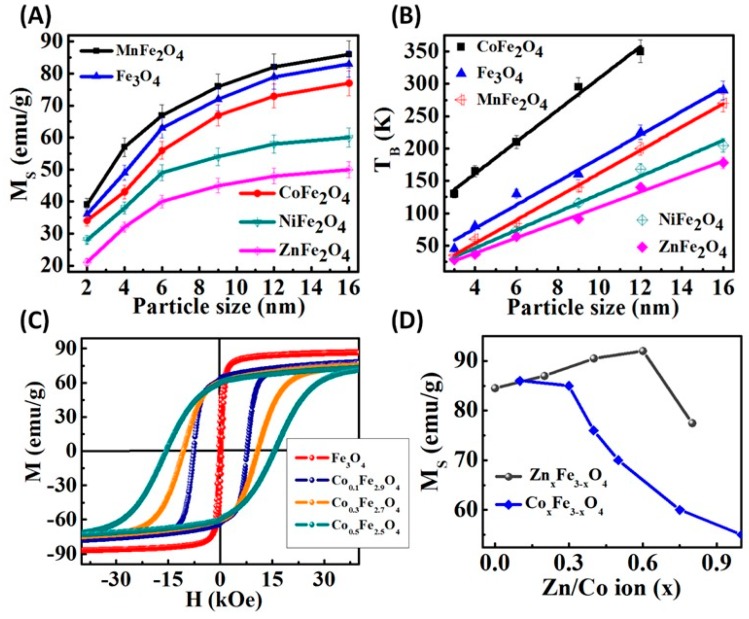
(**A**) Saturation magnetization vs. NPs size plot for _MFe_2___O_4__ NPs demonstrates the sharp increase of magnetization value with respect to NPs size [92]. (**B**) Blocking temperature vs. NPs size plot indicates a linear relationship between blocking temperature and NPs size [92]. (**C**) Hysteresis curves of Co*_x_*Fe_3-*x*_O_4_ nanoparticles with *x* = 0, 0.1, 0.3 and 0.5 at 10 K [100]. (**D**) Dependences of *M_S_* on the Zn and Co substitution content (*x*) in Fe_3_O_4_ nanoparticles [99,102].

Compositional modification in MNPs via the substitution of M^2+^ (M = Mn, Co, Ni and Zn) cations have significantly altered the magnetic properties. For example, the Fe_3_O_4_ NPs have an inverse spinel structure with Fe^2+^ and Fe^3+^ occupying octahedral ‘*O_h_’* sites aligned parallel to the external magnetic field and Fe^3+^ in the tetrahedral ‘*T_d_*’ sites aligned antiparallel to the field. Since the magnetic moment of Fe^3+^ and Fe^2+^ with a high spin state are 5μ_B_ and 4*μ_B_*, respectively, the total magnetic moment per formula unit (Fe^3+^)*_Td_*(Fe^2+^Fe^3+^)*_Oh_*O_4_ is approximately 4*μ_B_*. The substitution of M^2+^ cations with magnetic moment 5*μ_B_*(Mn), 3*μ_B_*(Co) and 2*μ_B_*(Ni) for *O_h_* Fe^2+^ leads to changes in the net magnetization per mass of magnetic atoms: 110 emu/g (MnFe_2_O_4_), 101 emu/g (FeFe_2_O_4_), 99 emu/g (CoFe_2_O_4_), and 85 emu/g (NiFe_2_O_4_), respectively [90]. The composition and size have been controlled precisely to understand their effect on magnetic properties (Figure 9A). As expected, the *M_S_* values of similar sized MFe_2_O_4_ (M = Mn, Fe, Co, Ni and Zn) nanoparticles strongly depend on the magnitude of the M^2+^ cation magnetic moment (*nμ_B_*, where *n* = 5, 4, 3, 2 and 0 for Mn, Fe, Co, Ni and Zn, respectively) [91]. For instance, the *M_S_* values of 6 nm size MnFe_2_O_4_ nanoparticles is 71 emu/g which reduces as per the periodic arrangement to 65, 55 and 50 for FeFe_2_O_4_, CoFe_2_O_4_ and NiFe_2_O_4_ NPs, respectively [92]. Interestingly, the ZnFe_2_O_4_ nanoparticles have saturation magnetization of 40 emu/g which indicates random magnetic ordering at the nanoscale (6 nm), which would otherwise be antiferromagnetic in the bulk regime [93]. Further, the *M_S_* values increase rapidly towards the bulk value as the nanoparticle size is increased. For example, the 2 nm MnFe_2_O_4_ nanoparticles have an *M_S_* value of 39 emu/g, which increases to 86 emu/g (close to the magnetization value of 89 emu/g of bulk MnFe_2_O_4_) [94,95] as the size is increased to 16 nm. This increasing trend in magnetization value is attributed to the decay in surface spin disorder effects with the increase of the particle size.

The blocking behavior of MFe_2_O_4_ NPs is also strongly related to the composition as the magnetocrystalline anisotropy varies with the spin-orbit coupling strength of M^2+^ cations. Figure 9B shows the size dependence blocking behavior of MFe_2_O_4_ NPs. The exchange coupled CoFe [92]. In contrast to the Fe_3_O_4_ nanoparticles, the *T_B_* value of CoFe_2_O_4_ nanoparticles is higher. The difference in *T_B_* values for the same size NPs of various transition metal (M^2+^) substituted spinel nanostructure is related to the magnetocrystalline anisotropy. In magnetite, the Fe^3+^ cations (orbital angular momentum L = 0) evenly occupy the tetrahedral (*T_d_*) and octahedral (*O_h_*) sites and while M^2+^ (Fe^2+^) cations are only located in the octahedral (*O_h_*) sites. Thus, the magnetocrystalline anisotropy arises from the spin-orbit coupling strength of M^2+^ cations and this strength increases in an order like Mn^2+^ < Fe^2+^ < Co^2+^ [96]. The stronger spin-orbit coupling of Co^2+^ ions lead to larger *T_B_* for CoFe_2_O_4_ NPs. Further, in contrast to other ferrites, CoFe_2_O_4_ NPs reveal a rapid increase of *T_B_* with size increase_,_ which confirms the hypothesis of larger anisotropy (L-S coupling) energy for CoFe_2_O_4_ nanoparticles.

Recent work has shown that because of the large magnetocrystalline anisotropy constant (*K* = 2 × 10^5^ J/m^3^) of cobalt, it can be used to effectively tune the magnetic properties through the controlled doping of Co in Fe_3_O_4_, MnFe_2_O_4_ and ZnFe_2_O_4_ nanoparticles [97,98,99]. A systematic adjustment of Co stoichiometry in Co_x_Fe_3-x_O_4_ (*x* = 0, 0.1, 0.3 and 0.5) NPs from 0 to 0.5 results in an increase in magnetic anisotropy and coercivity with maximum value reached *x* = 0.5 (see Figure 9C) [99,100]. The *M_S_* value of Fe_3_O_4_ nanoparticles is 86 emu/g, and it is decreased to 82, 78 and 77 emu/g, respectively with the increase of Co^2+^ ion concentration due to the smaller magnetic moment of Co^2+^(3*µ_B_*) as compared to Fe^2+^(4*µ_B_*) [91]. Figure 9D shows the typical variation of *M_S_* value with modulation of the Co and Zn content in Fe_3_O_4_ nanoparticles. Interestingly, with the increasing Zn content in Zn_x_Fe_3-x_O_4_ (*x* from 0 to 0.6) nanoparticles, the *M_S_* value increases (from 84.5 to 91.9 emu/g) and a further increase of Zn content, the *M_S_* value sharply decreases. The initial increase in *M_S_* with increasing Zn content is believed to be due to the cation distribution among *T_d_* and *O_h_*-sites in the spinel structure. When the nonmagnetic Zn^2+^ ion replaces the magnetic Fe^3+^ ions in *T_d_* –site the net magnetic moment between *O_h_* and *T_d_* sites increases up to *x* = 0.6 [101]. A further increase of Zn content above 0.6 can partially replace the Fe^2+^ ions and also impair the superexchange interaction between the magnetic ions at *O_h_* and *T_d_* sites, which causes a rapid decrease in the net magnetic moment.

**Figure 10:**
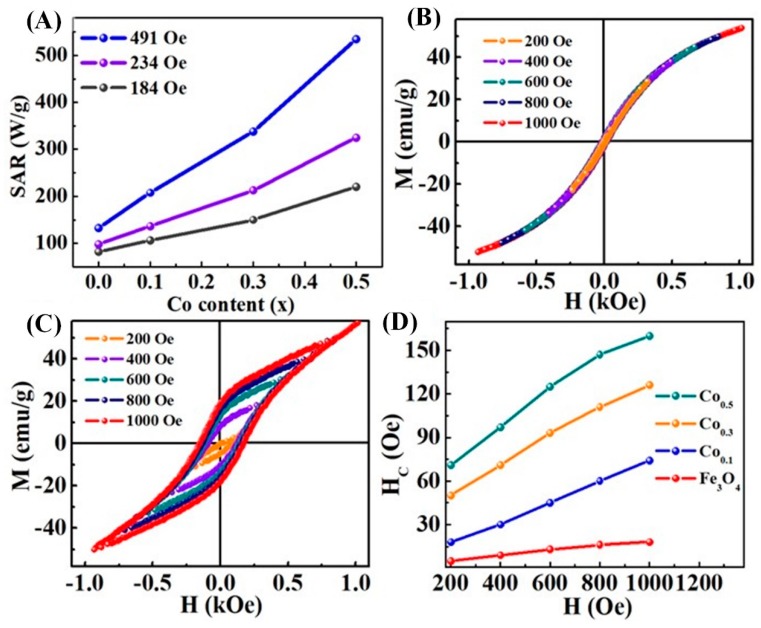
(**A**) Shows a plot of SAR as a function of Co content in Fe_3_O_4_ NPs across several values of applied ACMF field (184–491 Oe). (**B**,**C**) Show the room temperature minor M(H) loops at maximum magnetic field of 200, 400, 600, 800 and 1000 Oe for Fe_3_O_4_ and Co_0.5_Fe_2.5_O_4_ nanoparticles, respectively. (**D**) Shows the coercive field as a function of Co content in Fe_3_O_4_ NPs at various applied magnetic fields [100].

Like the magnetic properties, the heating efficiency of MNPs is strongly related to the composition. In order to demonstrate the effect of composition on hyperthermia properties, we take Co_x_Fe_3−x_O_4_ nanoparticles as an example, since Co strongly alter the magnetic anisotropy. Figure 10A shows the variation of SAR values for Co_x_Fe_3−x_O_4_ nanoparticles of size 12 nm. The obtained SAR value of pure Fe_3_O_4_ nanoparticles is 132 W/g at 265 kHz with an ACMF amplitude of 491 Oe and after being doped with Co^2+^ ions, the heating efficiency is greatly improved to 534 W/g. Besides, with the increase of the ACMF amplitude from 184 to 491 Oe, the SAR values of Fe_3_O_4_ and Co_0.5_Fe_2.5_O_4_ nanoparticles increase from 81 to 132 W/g and 220 to 534 W/g, respectively. As discussed above, the substitution of Co ions leads to an increase in *H_C_* at the cost of lower saturation magnetization. However, as nanoparticles with high *H_C_* and moderate *M_S_* are found to be the best heating agents, the modification of the composition proved to be effective in maximizing the SAR. As can be seen in Figure 10B–D, the SAR values are related to the hysteresis loop area. When the applied field keeps increasing, the *H_C_* and hysteresis loop area rapidly increase and strongly enhance the SAR value. It is important to note that, the Co-doped Fe_3_O_4_ NPs exhibit better heating activity compared to the pristine Fe_3_O_4_ and CoFe_2_O_4_ NPs prepared by a similar approach [97]. The effect of composition on the hyperthermia heating properties will be further discussed in the Section 3.4.

### 3.3. Shape Effects

Shape anisotropy is an extrinsic energy that comes from an induced demagnetizing field (*H_d_ = –N_d_M_S_*, where *N_d_* is the demagnetizing factor determined by the shape) of a magnetized body. In the case of spherical morphology, the surface poles are distributed over the surface such that there are none at the equator and most are at the poles. Hence the demagnetizing field is *H_d_* = –1/3*M_S_* and *N_d_* = 1/3 (for sphere). While, in the case of anisotropic morphology, the distribution of surface poles depends on the direction of magnetization. For example, in a prolate ellipsoid that is magnetized parallel to the major axis *c*, the free poles are farther apart, hence, *N_c_ <* 1/3 (as *H_d_* α 1*∕r*^2^). Similarly, if it is magnetized along axis *a*, the free poles are close to each other, hence, *N_a_ >* 1/3. The general expression of shape anisotropy constant (*K_sh_*) is *K_sh_ = M_S_^2^(N_a_ – N_c_)/2* [35,103]. A predominant effect of shape anisotropy on stoichiometry, coercivity and *M_S_* values has been observed in different anisotropic magnetic nanostructures [104,105,106,107,108,109,110]. 

**Figure 11:**
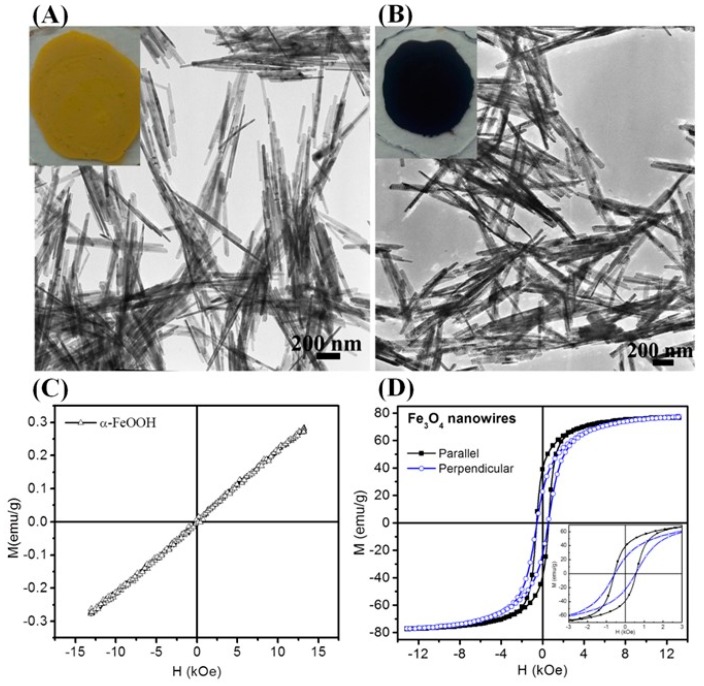
(**A**) TEM image of α-FeOOH NWs synthesized by hydrolysis of FeCl_3_ at 160 °C. Inset shows the α-FeOOH NWs powder produced by a single experiment. (**B**) TEM image of Fe_3_O_4_ NWs synthesized by annealing α-FeOOH NWs at 300 °C. Inset shows the color of Fe_3_O_4_ NW powder obtained after annealing α-FeOOH NWs. Room temperature hysteresis loops of (**C**) α-FeOOH NWs and (**D**) Fe_3_O_4_ NWs. The insert of D shows the low-field magnetization loops [111].

**Figure 12:**
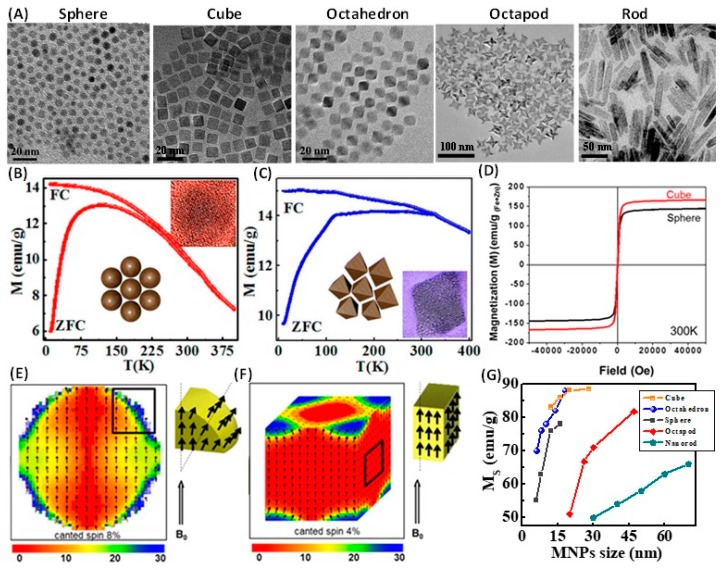
(**A**) TEM images of Fe_3_O_4_ spheres (8 nm), cubes (12 nm) [115], octahedrons (10 nm) [68], octopods [116] and rods [69] are obtained through thermolysis of iron salt. FC and ZFC magnetization curves of (**B**) spherical and (**C**) octahedral Fe_3_O_4_ nanoparticles at the applied field of 200 Oe [68]. (**D**) Room temperature M-H curves of cube and sphere-shaped Fe_3_O_4_ nanoparticles [2]. Simulated magnetic spin structures of (**E**) cube and (**F**) sphere. The color map indicates the degree of spin disorder in an external magnetic field, where red indicates ordered spins and blue indicates disorder spins. The right corners of parts E and F show their local surface spin arrangements [2]. (**G**) The variation of *M_S_* of different anisotropic Fe_3_O_4_ with increase of size [68,69,117].

Recently, Fe_3_O_4_ nanowires were synthesized via high-temperature reduction of α-FeOOH NWs in a fluidized bed reactor [111]. The removal of water molecules and the shearing of the oxide ion planes from AB to ABC stacking during phase transformation from the FeOOH to Fe_3_O_4_ make these Fe_3_O_4_ NWs porous (Figure 11A,B) [112]. The magnetization curves for Fe_3_O_4_ parallel to aligned NWs exhibited a *M_R_* of 0.51*M_S_* and *H_C_* of 583 Oe, respectively. When the samples were measured perpendicular to the alignment direction, remanence of *M_R_* = 0.30 *M_S_* was obtained, which indicates the influence of shape anisotropy [113,114]. A prominent effect of the MNPs shape on the surface anisotropy has also been observed in anisotropic-shaped nanoparticles. For example, the Verwey transition in Fe_3_O_4_ is a structural phase transition that is observed in the smallest octahedral NPs (6 nm) but does not even occur in the bigger size spherical NPs (13 nm) (Figure 12B,C) [68]. While the spherical-shape NPs shows the predictable superparamagnetic blocking behavior, which indicates the role of shape anisotropy on the stoichiometry. It is found that the facets of octahedral particles are consist of the most energetically stable [111] planes and the facets are protected against surface oxidation due to oleylamine coating. Thus, the surface anisotropy is significantly reduced, since the flat surface of the octahedron has fewer broken bonds and oxygen vacancies. A lower concentration of defects and almost no oxidized layer at the surface afford a better stoichiometry to the octahedral shape NPs and make the appearance of Verwey transition. In contrast, spherical NPs have highly disordered spins distributed across its outer surface which contribute to poor stoichiometry. As the Verwey transition is highly sensitive to stoichiometry, it is suppressed by the superparamagnetic blocking behavior.

The effect of shape anisotropy on ordering of surface atomic spins has also been well demonstrated by Cheon et al. [2]. Among a cube and a sphere with the same material volume and number of cations of Zn_0.4_Fe_2.6_O_4_ nanoparticles, the cube has greater *M_S_* (165 emu/g_(Fe+Zn)_) compared to that of the sphere (145 emu/g_(Fe+Zn)_) (Figure 12D). The cube has lower energy surface facets of family (100); in contrast, the surface of a spherical nanoparticle is constructed of different facets, which results in a larger surface spin disorder, hence higher surface anisotropy. A simulated result shows that the density of the disordered spins is higher in the sphere than in the cube (Figure 12E,F). The images are color-mapped according to the angle of the spin deviation versus the external magnetic field, red indicates ordered spins and blue indicates highly disordered spins. The disordered spins in blue are dominant at the corners of the cube, while they are broadly distributed at the surface of the sphere. The shape effect has also been noticed in Fe_3_O_4_ nanostructures of different shapes. The change in magnetization value with respect to shape and size of these nanostructures is summarized in Figure 12G. Among all these magnetic nanostructures, cube and octahedron exhibit enhanced *M_S_* values. The enhanced *M_S_* values are assumed to be due to anisotropic shape which lowers the surface spin disordered effect as explained in Zn_0.4_Fe_2.6_O_4_ nanocubes case [2,68]. The *M_S_* value of the rods is 50–65 emu/g. The low *M_S_* value is attributed to the surface spin canting or surface organic defective layer of the nanorods. 

**Figure 13:**
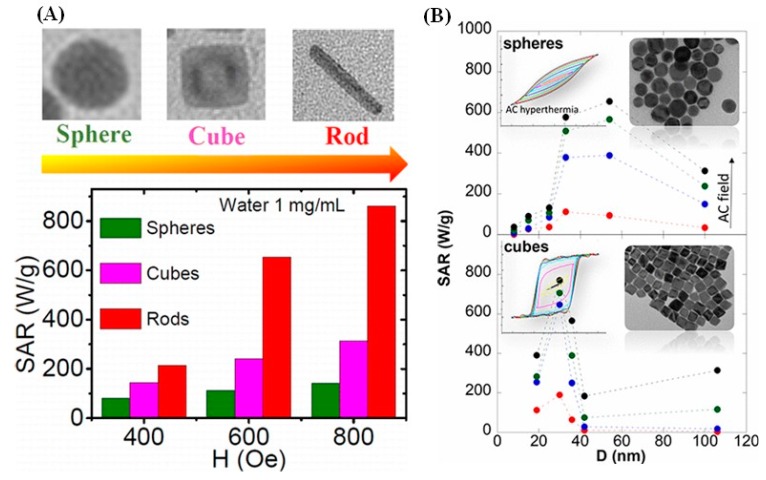
(**A**) SAR vs. field plot for the Fe_3_O_4_ spheres, cubes, and nanorods of nearly the same volume (∼2000 nm^3^) [89]. (**B**) SAR vs. size curves for the sphere and cube-shaped NPs measured in water (1 mg/mL) under AC magnetic fields of 0–800 Oe and at frequency of 300 kHz [118].

From the above discussion, we have seen that magnetic properties, particularly the *M_S_* and magnetic anisotropy strongly varied with the shape of the MNPs. Based on the superior magnetic properties, these anisotropic nanoparticles have also been shown to be a better heating candidate compared to the spherical counterpart. Recently, a large improvement in SAR has been reported for cubes, octopods, octahedrons, plates and rods, as a result of their improved magnetic anisotropy. Taking into account the proven advantages of high-aspect-ratio Fe_3_O_4_ nanowires/nanorods over their spherical and cubic counterparts, such as larger surface area, enhanced blood circulation time, and prolonged retention in tumors, Das et al. demonstrated higher heating activity in Fe_3_O_4_ nanorods of aspect ratio from 6 to 11 [89]. Figure 13A shows a comparison between spherical and cubic NPs and compared their SAR values with those of the Fe_3_O_4_ nanorods. Interestingly, the nanorods have higher SAR values than those obtained for the sphere and cube-shaped NPs, particularly in the high field region (>600 Oe). At 800 Oe the SAR value is 862 W/g for the nanorod, while it is only about 140 and 314 W/g for the spheres and cubes, respectively. The nanorods exhibit superior heating efficiency because of their larger *K_eff_* values, related with their shape anisotropy. Further, increasing the aspect ratio of the nanorods from 6 to 11 improves the SAR by 1.5 times. 

From the above discussion we have already seen a higher heating activity for cubes than the spheres. For further understanding, Nemati *et al.* demonstrated a comparison between sphere and cube-shaped nanoparticles in a wide range of sizes, ∼10–100 nm (Figure 13B) [118]. The spheres show negligible SAR for sizes less than 13 nm, but then sharply increases beginning near 26 nm until it reaches a broad maximum of ~650 W/g at 800 Oe around 52 nm. The very low SAR values observed by the small-sized nanospheres is related to their broader size distribution. However, for the cube-shaped NPs, the heating efficiency shows a very different trend; the size evolution of the SAR surprisingly increases at 30 nm up to ∼800 W/g at 800 Oe and then decays immediately with increasing size, reaching a minimum for the 42 nm cubes. 

### 3.4. Optimization of Intraparticle and Interparticle Interactions

#### 3.4.1. Effect of Interparticle Interactions

**Figure 14:**
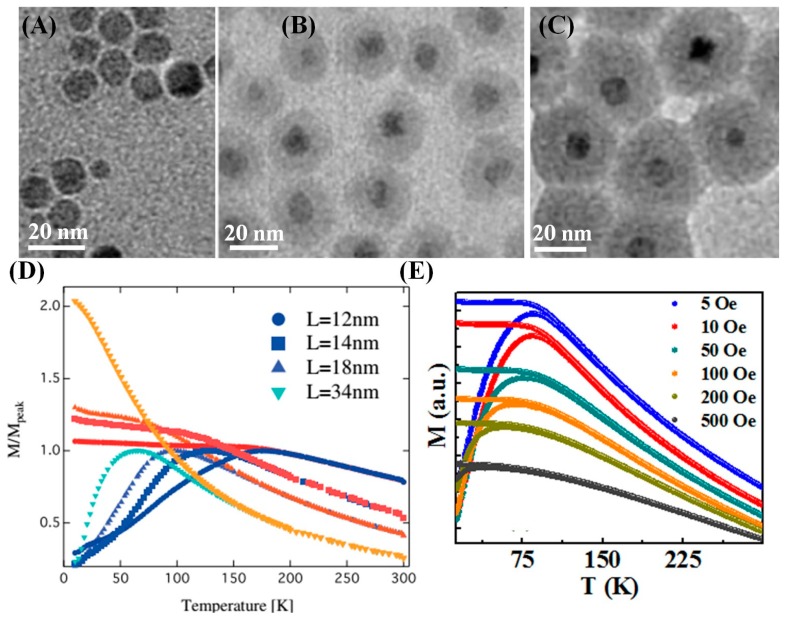
(**A**–**C**) TEM images of γ-Fe_2_O_3_ NPs (average diameter = 11 nm), γ-Fe_2_O_3_/SiO_2_ NPs with SiO_2_ layer thickness, L = 26 nm, and 34 nm, respectively. (**D**) Temperature-dependent magnetization of the γ-Fe_2_O_3_/SiO_2_ NPs with SiO_2_ layer thickness, L = 12, 14, 18, and 34 nm under ZFC and FC conditions [125]. (**E**) The ZFC and FC magnetization are measured at the indicated field values for 4 nm Fe_3_O_4_ nanoparticles [43].

It is known that in concentrated particle systems the magnetic behavior of the particles is significantly influenced by interparticle interactions. The magnetic properties of such MNP systems are studied using a frozen state of ferrofluid where MNPs are dispersed in solvent or by embedding the MNPs in a solid matrix [43,119,120,121]. Multiple studies report that the anisotropic-energy barrier in interacting nanoparticle systems increases with the increase of interparticle interaction, i.e., by decreasing the separation distance among the MNPs. Thus, the blocking temperature of strongly interacting MNP systems is higher than that in the corresponding isolated MNP system [122,123,124]. For example, Fe_3_O_4_/SiO_2_ core-shell nanoparticles with different shell thicknesses (Figure 14A–C) are prepared to understand the effect of interparticle interactions on the magnetic properties [125,126,127]. As the shell thickness decreases, the influence of interparticle dipolar interaction becomes apparent and quasi-magnetostatic states like superspin-glass (SSG) and super-ferromagnetic are observed. Figure 14D shows the temperature-dependent magnetization of Fe_3_O_4_/SiO_2_ core-shell nanoparticles. With increasing the SiO_2_-shell thickness on the MNPs, the *T_B_* is lowered and the field-cooled magnetization (*M_FC_*) at low temperature becomes pronounced. These features are particularly observed in conventional superparamagnetic NPs systems in which the interparticle interaction is negligible. In the most concentrated sample, L12, the *M_FC_* at low-temperature displays no increase but rather a slight decrease, which is a superspin-glass like behavior. The flat nature of *M_FC_* curves can be more prominent in dense nanoparticle assemblies. This cooperative magnetic behavior can also be described from the field dependence of *M_ZFC_* and *M_FC_* measurements. When the applied magnetic field increases, the superspin glass transition peak is prominent and changes to a plateau like shape as observed in 4 nm Fe_3_O_4_ nanoparticle assemblies (Figure 14E). Further, the temperature at which the irreversibility between *M_ZFC_* and *M_FC_* curves appears shifts towards lower values with the increases in magnetic field (5–500 Oe), a characteristic of spin-glass systems. 

**Figure 15:**
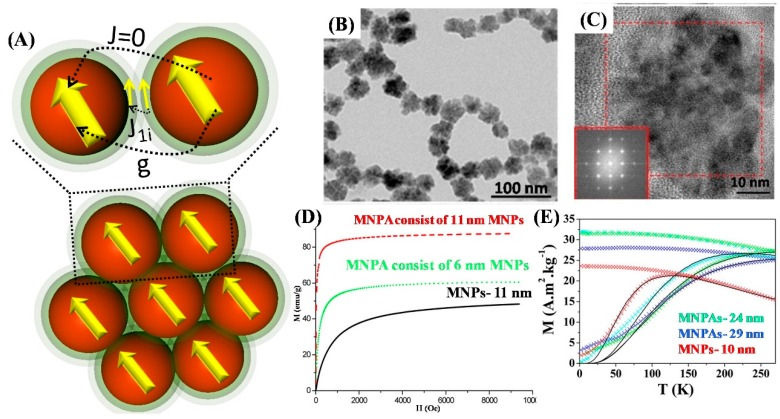
(**A**) Schematic illustration of the supermoment structure and the intra-particle as well as inter-particle interactions in a nanoparticle assembly. Outermost light green color: Surfactant polymeric layer [70]. (**B**) TEM and (**C**) HR-TEM micrographs and Fourier transformation of flowerlike nanoparticles [24]. (**D**) Magnetization curves of 11 nm-sized nanoparticles synthesized via coprecipitation (green dotted line), 6 nm nanoparticles synthesized in diethylene glycol (black solid line) and 11 nm nanoparticle assemblies synthesized in a mixture of diethylene glycol and *N*-methyl diethanolamine (red dashed line). (**E**) ZFC-FC magnetization curves taken with an applied field of 50 Oe: 24 nm (cyan), 29 nm (blue)-sized nanoparticles assembly and 10 nm (red)-sized nanoparticles [25].

The inter-particle interaction can become more pronounced in the case of nanoparticle assemblies, superlattices or aggregates. In fact, these secondary nanostructures (magnetic nanoparticle nano-assemblies, noted MNPAs), the dipolar coupling and exchange-coupling (Figure 15A) between the neighboring nanocrystals can be comparable to the magnetic anisotropy energy. Therefore, the energy barrier of individual nanoparticles is strongly impacted by exchange and dipolar interactions [70]. The increase in the energy of the barrier shifts the superparamagnetic blocking temperature of MNPAs to a higher temperature than that of the isolated nanoparticles. Moreover, the MNPAs also exhibits a phase transition, from a superparamagnetic state to a collective magnetic behavior at low temperature. The quasi-magnetostatics state is caused by the frustration of interparticle interactions that are induced due to randomness in nanoparticle positions and anisotropy-axes orientations. The collective behavior is called superspin-glass and has been recently observed in Fe_3_O_4_, γ-Fe_2_O_3_ and CoFe_2_O_4_ nanoparticle nano-assemblies [7,8,128]. For example, Lartigue *et al.* synthesized different sized nanoparticle assemblies (19.7, 22.2, 24.0, and 28.8 nm) of γ-Fe_2_O_3_ nanoparticles (10 nm) using a single-step high-temperature hydrolysis approach (Figure 15B,C) [24,25]. These MNPAs present a bulk-like *M_S_* value (80 emu/g for maghemite), while the 10 nm nanoparticles show a 30% reduction of *M_S_* value with respect to the bulk value (Figure 15E). The susceptibility of the MNPAs is higher than that of the 10 nm nanoparticles and increases with the increase in the sizes of the MNPAs. The higher *M_S_* values in the case of the MNPAs is believed to be due to the existence of exchange interactions between the surface atoms of neighboring nanoparticles. The exchange-coupling between closely packed nanoparticles with different orientations of easy axes can certainly result in a rotation of the spin structure to an aligned ordered structure at the interface as shown in Figure 15A. The collective behavior of spins at the interface lowers the surface anisotropy and also results in high magnetic susceptibility (Figure 15D). Further, in comparison to nanoparticles, a large increase in the blocking temperature by 150 K is observed for MNPAs (Figure 15D). In addition, as expected the FC curve flattens out below *T_B_*. The *M_FC_* remains flat below *T_B_*, indicating the presence of a spin-glass-like state due to magnetic interactions among the nanoparticles within the assembled structure [8].

**Figure 16:**
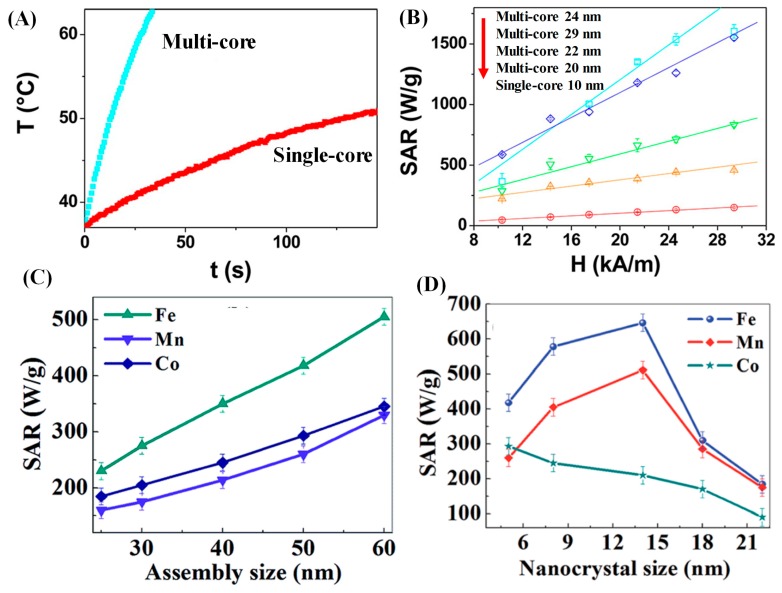
A comprehensive demonstration of the optimization of hyperthermia heating efficiency of MFe_2_O_4_ MNPAs by controlling size, composition, and magnetic coupling among the nanocrystals within the nanoassembly. (**A**) Heating curves for multi-core and single-core Fe_3_O_4_ nanostructures are recorded under an ACMF of amplitude 29 kA/m and at frequency 520 kHz [25]. (**B**) SAR values as a function of the ACMF amplitude at frequency *f* = 520 kHz for multi-core 24 nm (cyan), multi-core 29 nm (blue), multi-core 22 nm (green), multi-core 20 nm (orange), and single-core 10 nm (red) [25]. (**C**) The SAR as a function of nanoassembly size of MFe_2_O_4_ (M = Mn, Fe and Co) with nanocrystal size 4 nm [70]. (**D**) The SAR as a function of nanoassembly size of MFe_2_O_4_ with variation of nanocrystal size from 6 to 21 nm [70].

Recently, the MNPAs, also named nanoflowers, nanocluster, and nanocrystal assemblies have shown good heating efficiency due to cooperative magnetism among nanocrystals within the multi-domain nanostructure. Lartigue et al. demonstrated a comparison of heating performance of MNPAs (multi-core nanostructures) with the single-core nanoparticles [25]. Under field conditions of 29 kA/m and 520 kHz, the temperature increased at a rate of 1.04 °C/s for MNPAs, while that for single-core nanoparticles showed only 0.15 °C/s (Figure 16A). Compared to the rest of the size-sorted multi-core nanostructures, the largest sized sample showed the highest SAR (Figure 16B). More importantly, the SAR of the multi-core nanoparticles is much higher than that reported for single-core nanoparticles. In fact, they are among the best heating materials reported so far for iron oxide nanoparticles. A similar trend is also observed in MFe_2_O_4_ (M = Mn, Fe, Co and Ni) nanoparticle nanoassemblies produced via thermal decomposition of metal chloride in ethylene glycol in the presence of ethylenediamine. The heating performance of the MFe_2_O_4_ MNPAs as a function of nanoassemblies and nanocrystals size are measured at a frequency of 247 kHz and ACMF amplitude of 310 Oe. The SAR values of all the MFe_2_O_4_ MNPAs display an increase trend with the increase in size of MNPAs while keeping the nanocrystal size same (Figure 16C). The results from the magnetic characterization verify the enhancement of *M_S_* value with the increase of MNPAs size and the presence of collective magnetic dynamics, i.e., the collective Néel relaxation of nanocrystals within the assembly. Thus, the increase of SAR with MNPAs size is ascribed to the cooperative Néel relaxation and the high particle magnetic moment. An important composition effect has also been noticed in MFe_2_O_4_ MNPAs. In contrast to CoFe_2_O_4_ (high *K_eff_*) and MnFe_2_O_4_ (high *M_S_*), the Fe_3_O_4_ MNPAs have an exclusively high SAR value in all the size ranges (Figure 16D). From the Section 2, we know that high *K_eff_* leads to a shift in the critical particle size associated with the maximum heating to a lower particle size value (as critical radius ‘*R_0_’* corresponding to maximum heating *R_0_* ∝ 1/*K_eff_*). In this case, the calculated critical nanocrystal size corresponding to maximum SAR are 6, 15 and 27 nm for CoFe_2_O_4_, Fe_3_O_4_ and MnFe_2_O_4_ nanocrystals, respectively. Thus, the MnFe_2_O_4_ and Fe_3_O_4_ MNPAs exhibit a maximum heating at the nanocrystalline size of 14 nm, i.e., 80 nm sized MNPAs while CoFe_2_O_4_ MNPAs displays the maximum heating activity for 4 nm size nanocrystals, i.e., 50 nm sized MNPAs.

In order to demonstrate the efficacy of magnetic nanoparticles solution for cancer treatment, the hyperthermia heating properties of magnetic nanoparticles are measured in the cellular environment. The study demonstrated that irrespective of size, shape, and compositions of the magnetic nanoparticles, the heating activity rapidly dropped following the internalization [129]. More importantly, the SAR values in the cellular environments are half than those obtained in the solution. The low SAR values are related to the cellular confinement effect, which completely inhibited the Brownian relaxation. In contrast to the superparamagnetic nanoparticles, the heating properties of ferromagnetic nanoparticles are significantly impacted after contact with cells. Despite the above issues, several research works have demonstrated that the nanoparticles morphology, magnetic and chemical property modification can address the above challenges [130,131,132,133].

#### 3.4.2. Effects of Intraparticle Interactions

**Figure 17:**
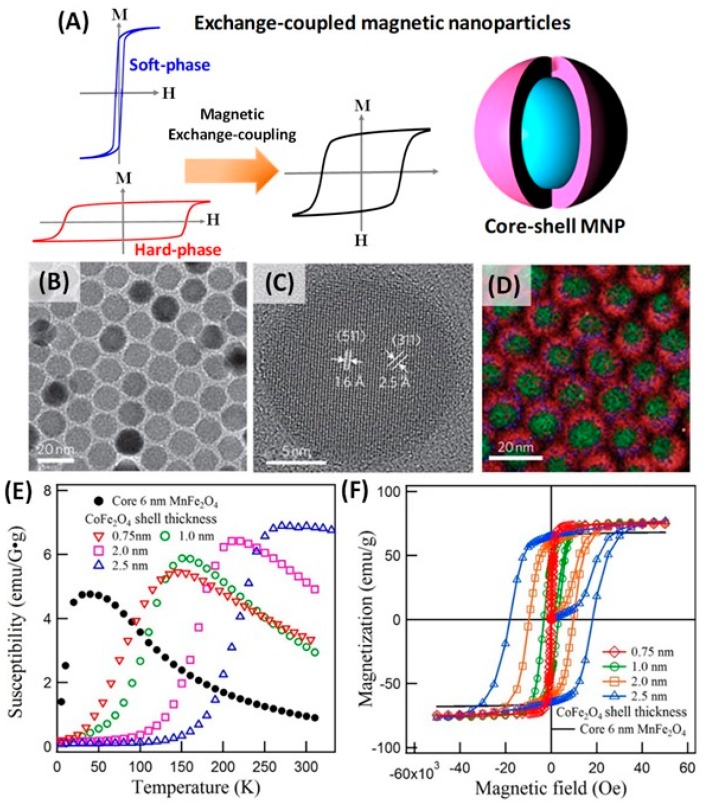
(**A**) A schematic depiction of the hysteresis loops for soft and hard nanoparticles and their possible combinations: exchange-coupled core-shell nanoparticles. (**B**) TEM image, (**C**) high-resolution TEM image of 15 nm CoFe_2_O_4_/MnFe_2_O_4_ core–shell NPs and (**D**) Overlay of electron energy-loss spectrum mapped images of Co (green), Fe (red) and Mn (blue) [31]. (**E**) Temperature dependent susceptibility under 100 Oe field and (**F**) hysteresis loops at 5 K for MnFe_2_O_4_/CoFe_2_O_4_ core–shell nanoparticles with variation of shell thickness as 0, 0.75, 1.0, 2.0 and 2.5 nm [139].

Exchange coupled core-shell nanoparticles with soft core (low anisotropy, high magnetization) and hard shell (high anisotropy) or vice versa is a prominent example of an intraparticle interacting system. The core-shell nanoparticles exhibit improved magnetic properties, but more importantly they produce an intermediate magnetic anisotropy and saturation magnetization compared to both the constituents (Figure 17A) [124,125,126,127,128,129,130,131,132,133,134,135,136,137,138]. In the nanocomposite system, the magnetization of the soft magnetic phase is able to rotate coherently with that of the hard-magnetic phase, thus allowing us to utilize the advantages of soft and hard magnetic phases. 

Recently, the exchange-coupled core-shell nanoparticles have been explored to modulate magnetic properties of the spinel ferrite and have shown substantial importance in biomedical applications [139,140]. By picking cobalt ferrites and/or other soft magnetic ferrites as starting materials, a core–shell structure with distinctly controllable uniformity and size dimensions can be acquired. In a typical synthesis of CoFe_2_O_4_/MnFe_2_O_4_ core–shell nanoparticles, the CoFe_2_O_4_ NPs dispersed in hexane were injected into the mixture of MnCl_2_, Fe(acac)_3_, oleic acid and oleylamine [134]. The reaction was then refluxed at 365 °C for 1 h and MnFe_2_O_4_ was overgrown onto the surface of the CoFe_2_O_4_ seeds to form a core–shell structure. The same approach can be extended to synthesize other core-shell nanoparticles such as CoFe_2_O_4_/Fe_3_O_4_, CoFe_2_O_4_/ZnFe_2_O_4_ and MnFe_2_O_4_/ZnFe_2_O_4_. The core-shell structures can be confirmed from the high-resolution TEM and electron energy-loss spectrum (EELS) mapping analysis as shown in Figure 17B–D. Figure 17D shows superimposed EELS mapped images of CoFe_2_O_4_/MnFe_2_O_4_ nanoparticles. In this figure Co, Fe, and Mn are color-coded in green, red and blue respectively. While Co is present only in the core region of each nanoparticle, Fe is distributed throughout the nanoparticle, and Mn only on the shell. The exchange-coupled magnetism is confirmed from the enhanced coercivity and magnetic anisotropy values. At 5 K, the *H_C_* for the CoFe_2_O_4_/MnFe_2_O_4_ nanoparticles falls between the values for CoFe_2_O_4_ and MnFe_2_O_4_ nanoparticles: *H_C_* (CoFe_2_O_4_/MnFe_2_O_4_) = 2.53 kOe, *H_C_* (CoFe_2_O_4_) = 11.6 kOe and *H_C_* (MnFe_2_O_4_) = 0 Oe. Song et al. synthesized MnFe_2_O_4_/CoFe_2_O_4_ core–shell NPs with a core diameter of 6 nm, and the shell thickness can be precisely controlled from 0.5 to 3 nm [139]. The relationship of the shell thickness with the blocking temperature and the coercivity of MnFe_2_O_4_/CoFe_2_O_4_ core–shell nanoparticles with changing the shell thickness from 0.5 to 3 nm are shown in Figure 17E,F. The blocking temperature and coercivity in the core–shell nanoparticles increase as the shell thickness increases. 

**Figure 18:**
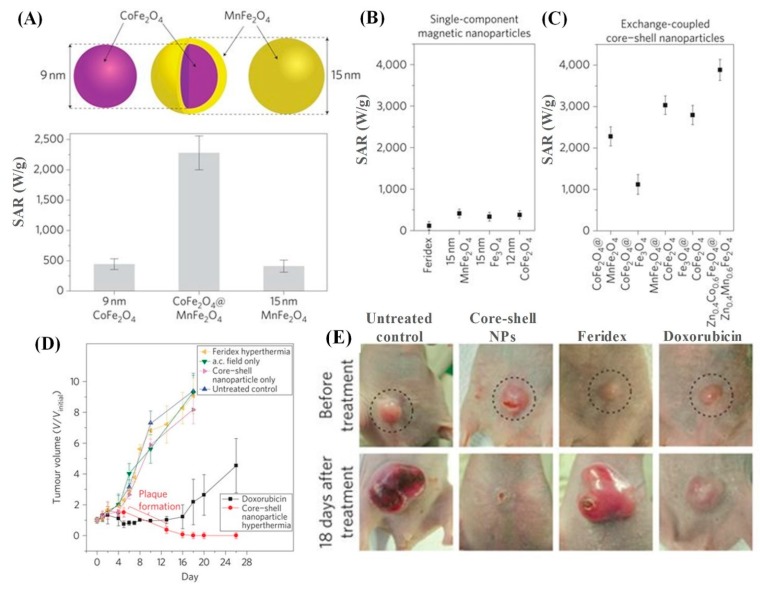
(**A**) Schematic view of 15 nm CoFe_2_O_4_/MnFe_2_O_4_ core/shell MNPs and its SAR value in comparison with the values for its components (9 nm CoFe_2_O_4_ and 15 nm MnFe_2_O_4_). (**B**) SAR values of single-component magnetic NPs and (**C**) the various of SAR values of combinations of core–shell NPs. (**D**) Plot of tumor volume (*V*/*V*_initial_) versus days after hyperthermia treatment with the single-component and core–shell NPs. In the group treated with core–shell MNPs-based hyperthermia, the tumor was completely eliminated in 18 days. (**E**) Nude mice xenografted with cancer cells (U87MG) before treatment (upper row, dotted circle) and 18 days after treatment (lower row) with untreated control, CoFe_2_O_4_@MnFe_2_O_4_-based hyperthermia, Feridex-based hyperthermia and doxorubicin, respectively [31].

From the above discussion we have noticed that the magnetic anisotropy, which plays a critical role in hyperthermia heating can be tuned by controlling the core-shell dimensions. Theoretical analysis discussed in the Section 2.2 confirmed that the SAR of superparamagnetic NPs could be optimized when the *K_eff_* will be in the range from 0.5 × 10^4^ to 4.0 × 10^4^ J/m^3^ (i.e., $\;\mathrm{the}\;\mathrm{case}\;\mathrm{of}\;2\pi f\sim \tau^{-1}$), but the *M_s_* factor must also be considered in attempting to maximize the SAR. To attain the right *K_eff_* and *M_S_* combination, the exchange-coupled core-shell nanoparticles of hard/soft magnetic ferrites are important. A typical example is the core/shell nanoparticles composed of magnetically hard CoFe_2_O_4_ (*K* = 2 × 10^5^ J/m^3^) core and magnetically soft MnFe_2_O_4_ shell (*K* = 3 × 10^3^ J/m^3^) [31]. The exchange coupled CoFe_2_O_4_/MnFe_2_O_4_ nanoparticles (~15 nm in size) preserved the superparamagnetism at room temperature and showed a *K_eff_* of 1.5 × 10^4^ J/m^3^, which is in the best possible *K_eff_* range. As a result, the core/shell nanoparticles exhibited 5 times higher SAR value of 2280 W/g of magnetic elements compared to single component NPs (443 W/g for the CoFe_2_O_4_ NPs and 411 W/g for the MnFe_2_O_4_ NPs) in an ACMF of *H_ac_* = 37.3 kA/m and *f* = 500 kHz (Figure 18A). In addition, the SAR values can be tuned by varying the combination of the core and shell components as shown in Figure 18B,C. The magnetic coupling of core and shell components provides *K_eff_* values of ∼1.5 × 10^4^ to 2.0 × 10^4^ J/m^3^, which fits in the optimal *K_eff_* range. Interestingly, the Zn_0.4_Co_0.6_Fe_2_O_4_ /Zn_0.4_Mn_0.6_Fe_2_O_4_ NPs show an extremely high *M_S_* of 150 emu/g of magnetic elements and when used for hyperthermia heating it shows SAR around 3886 W/g of magnetic elements. This is 1.7 times greater than that for CoFe_2_O_4_/MnFe_2_O_4_ and 34 times larger than that for Feridex, the commercial iron-oxide magnetic nanoparticle (115 W/g). While the SAR values of core-shell nanoparticles are comparable to those of ferromagnetic and anisotropic nanoparticles, the advantage of core-shell nanoparticles over the latter’s is that they exhibit superparamagnetism at room temperature, an important property for the clinical applications. 

The in vivo hyperthermia treatment is performed by injecting 75 μg of the CoFe_2_O_4_/MnFe_2_O_4_ core/shell nanoparticles into the U87MG human brain cancer cells in mice and then applying the ACMF for 10 min. The tumor volume continuously shrinks with time, which is noticed for the core/shell NPs (Figure 18D). In fact, the tumor is eliminated after an 18 days hyperthermia treatment with the formulated core/shell NPs solution. While, the tumors volume did not shrink by using Feridex (commercial ferrofluid), or chemotherapeutic doxorubicin cancer drug under the similar treatments, but displayed growth behavior like the untreated control (Figure 18E). In short, these core-shell ferrites can be an effective new nanoscale tool beneficial for a variety of systems that rely on heat induction, including hyperthermia therapy and other advanced nanobiotechnology applications such as on-demand drug release and thermal activation of metabolic pathways within a single cell.

## 4. Conclusions 

In this review, the close correlations are discussed between the ferrite nanoparticles properties (magnetic and morphological) and the hyperthermia performance. The size, composition, shape, inter-particle interaction and inter-phase exchange coupling are utilized to tune the magnetic properties and consequently the heating performance of MNPs. The control over size from a nanometer to a submicron length scale and incorporation of different transition metal ions lead to the improvement of magnetic properties (*M_S_* and *K_eff_*). The size dependence of magnetic and inductive heating properties reveals that the large size Fe_3_O_4_ nanoparticles (>16 nm) are in ferromagnetic regime which gives extremely high SAR value above 800 W/g. Alternatively, the *M_S_* and *H_C_* are improved by introducing the shape anisotropy. Particularly, the iron oxide nanorods and nanocubes exhibit unprecedented heating ability compared to spherical nanoparticles of equivalent material volume. Besides, the collective magnetic response is achieved by modifying interaction strength between the nanoparticles. The nanostructures composed of number of magnetic domains such as, superlattice, nanoflowers and nanocrystal assemblies have also demonstrated efficient heating effect due to the cooperative magnetism among nanocrystals. Hard and soft ferrite core-shell architecture with strong exchange coupling optimizes *K_eff_* to an intermediate value of the soft and hard ferrites. The intermediate *K_eff_* value makes the nanoparticles relax with a frequency of ACMF, which enhances the SAR value to a surprisingly high value of 3886 W/g of magnetic elements. The prominently high SAR values obtained via optimization of magnetic performance demonstrate the promising future of iron oxide nanoparticles for biomedical applications. Nevertheless, a long-term stability, toxicological impact, site-specific internalization and metabolism of the magnetic nanoparticles are still open questions and more extensive research is needed. Finally, new types of smart magnetic nanoparticles with multifunctional properties are still needed for cancer theranostics. A reproducible and large-scale synthesis approach of magnetic nanoparticles also needs to be established for the industry perspective. Although the described magnetic nanoparticles are functionalized with a suitable surfactant for the hyperthermia application, but it can also be further functionalized or decorated with various functional materials such as targeting molecules, biomarker/quantum dots, radioisotopes and drugs. The multifunctional magnetic nanoparticles will facilitate early diagnosis with multimodal imaging and simultaneous therapy. 

## Figures and Tables

**Table 1:** *T_C_*, *M_S_* and SAR values of different mixed ferrite nanoparticles.

Compound	MNPs Size	*T_C_*	*M_S_*	SAR	*Ref.*
Mg_1.37_Fe_1.26_Ti_0.37_O_4_	>100 nm	46 °C	8 emu/g	3.9 W/g	[61]
Zn_0.9_Fe_2.1_O_4_	11 nm	93 °C	12 emu/g	36 W/g	[58]
MnFe_1.4_Ag_0.6_O_4_	4–7 nm	87 °C	~15 emu/g	-	[62]
Zn_0.6_Cu_0.4_Fe_2_O_4_	460 nm	32 °C	-	-	[59]
Mn_0.8_Zn_0.2_Fe_2_O_4_	20–25 nm	42–45°C.	67 emu/g	110 W/g	[63]
Zn_x_Fe_3-x_O_4_ (0.01 ≤ x ≤ 0.8)	3–10 nm	~125 °C	35–65 emu/g		[60]
